# Impact of antiretroviral treatment on height evolution of HIV infected children

**DOI:** 10.1186/s12887-019-1663-8

**Published:** 2019-08-17

**Authors:** Patrinee Traisathit, Saïk Urien, Sophie Le Coeur, Sakulrat Srirojana, Noppadon Akarathum, Suparat Kanjanavanit, Chaiwat Ngampiyaskul, Sawitree Krikajornkitti, Nicole Ngo-Giang-Huong, Marc Lallemant, Gonzague Jourdain

**Affiliations:** 1 Department of Statistics, Faculty of Science, Chiang Mai University, Chiang Mai, Thailand; 2 Pediatric and perinatal pharmacology, Université de Paris, Paris, France; 3 Unité de Recherche Clinique Necker Cochin, AP-HP, Hôpital Tarnier, Paris, France; 4 CIC1419 INSERM, Cochin-Necker, Paris, France; 5 Institut de recherche pour le développement (IRD) UMI 174-PHPT, Marseille, France; 6 Faculty of Associated Medical Sciences, Chiang Mai University, Chiang Mai, Thailand; 7 Institut d’Etudes Démographiques, Paris, France; 8 Kalasin Hospital, Kalasin, Thailand; 9 Sanpatong Hospital, Chiang Mai, Thailand; 10 Nakornping Hospital, Chiang Mai, Thailand; 11 Prapokklao Hospital, Chanthaburi, Thailand; 12 Samutsakhon Hospital, Samutsakhon, Thailand; 13 Harvard T.H. Chan School of Public Health, Boston, MA USA

**Keywords:** Asia, antiretroviral therapy, catch-up growth, height-growth velocity, HIV-infected children, Thailand

## Abstract

**Background:**

Antiretroviral treatment (ART) has been shown to have a beneficial effect on the weight evolution but its effect on height remains unclear. We described patterns of height evolution and identified predictors of catch-up growth in HIV-infected children on ART.

**Methods:**

To describe the height evolution from birth to adulthood, we developed a nonlinear mixed effect model using data from perinatally HIV-infected children who initiated ART from 1999 to 2013 in a prospective cohort study in Thailand. The main covariates of interest were: sex, ART regimen (dual nucleoside reverse-transcriptase inhibitor, non-nucleoside reverse transcriptase inhibitor (NNRTI)-, or protease inhibitor (PI)-based), baseline CD4 percentage, HIV-RNA load and CDC HIV Classification stage and occurrence of AIDS-defining events.

**Results:**

A total 477 children (43% boys) contributed 18,596 height measurements over a median duration of 6.3 years on ART (interquartile range, 3.0 to 8.3). At ART initiation, median age was 6.2 years (1.8 to 9.6), 16% of children were underweight (weight-for-age z-score < − 2), 49% presented stunting (height-for-age z-score < − 2), and 7% wasting (weight-for-height z-score < − 2). The most frequent regimen at ART initiation was NNRTI-based (79%). A model with 4 components, birth length and 3 exponential functions of age accounting for the 3 growth phases was developed and show that the height-growth velocity was inversely associated with the age at ART initiation, the adult height was significantly lower in those who had experienced at least one AIDS-defining event while, as expected, the model found that adult height in females was lower than in males. Age at ART initiation, type of ART regimen, CDC stage, CD4 percentages, and HIV-RNA load were not associated with the final height.

**Conclusions:**

The younger the children at ART initiation, the greater the effect on height-growth velocity, supporting the World Health Organization’s recommendation to start ART as early as possible. However, final adult height was not linked to the age at ART initiation.

**Electronic supplementary material:**

The online version of this article (10.1186/s12887-019-1663-8) contains supplementary material, which is available to authorized users.

## Background

HIV infection in children has been associated with growth delays in terms of weight (wasting and underweight) and height (stunting) [1–9]. Antiretroviral treatments (ART) have been shown to have a positive impact on the evolution of the anthropometric parameters [8–13], but studies have mostly focused on the improvement of the weight-for-age z-score (WAZ) [9, 14–18]. Results of the several studies analyzing the effect of ART on height-for-age z-score (HAZ), a better indicator of the general development of children in the long term [19–23] are conflicting, some suggesting a favourable effect [24–27] and others not [28–32]. These discrepancies may be explained by some methodological issues: data were sometimes collected among a small numbers of children, with limited number of height measurements and growth response analysed only up to 12 months after ART initiation [28–34].

In the present study, we used data from a relatively large cohort of children born with HIV, followed for more than 6 years and with frequent height measurements. We were able to develop a mathematical function that describes the height evolution of children from birth to adulthood. This allowed to analyse the effect of ART on the growth of children, taking into account other factors that could influence it such as sex, clinical, virological and immunological status at the time of treatment initiation, as well as initial ART regimen and the occurrence of AIDS-defining events during the follow-up.

## Methods

### Patients and follow-up

We used data from all HIV-infected children who started ART within the Program for HIV Prevention and Treatment (PHPT) cohort study initiated in 1999 in 40 hospitals in Thailand (Clinicaltrial.gov: NCT00433030). Children’s anthropometric parameters were measured at enrolment and at each monthly visit during the first 2 years of follow up and every 6 months thereafter. HAZ, WAZ and weight-for-height (WHZ) were computed using the reference growth curves for Thai children updated in 2000 [35]. CD4 percentage, HIV-RNA viral load and Centers for Disease Control and Prevention HIV classification (CDC stage) [36, 37] were assessed at enrolment and every 6 months thereafter. AIDS-defining events [36, 37] were reported at the time of their occurrence.

### Descriptive statistics at ART initiation

Continuous variables were described using median and interquartile ranges (IQR), and categorical variables were presented as frequencies and percentages. HAZ was categorized as <− 3 SD, − 3 to <− 2 SD, − 2 to <− 1 SD and ≥ − 1 SD. HAZ categories at ART initiation were compared using chi-square test for categorical variables – sex, type of ART regimen, CDC stage, WAZ and WHZ—, and Kruskal-Wallis test for continuous variables –age, CD4 percentage and viral load. Chi-square test was used to assess the association between the occurrence of AIDS-defining events (ADEs) and HAZ at last follow-up visit.

### Modelling strategy and data analysis

We developed an empirical nonlinear mixed effect model to describe each child’s final height (*HT*) using Monolix (version 4.1.4) nonlinear mixed effect modelling program (http://lixoft.com/downloads/) [38]. Firstly, a height versus age scatter plot was drawn. As expected, the curve was non-linear. Thereafter, a log (Height) versus age showed that the curve could be roughly described by 3 successive straight segments defining 3 growth phases. A sum of 3 exponential functions was then used for describing growth as a function of age. Parameters were estimated separately for males and females by computing the maximum likelihood estimator of the parameters without any approximation of the model (no linearization) using the stochastic approximation expectation maximization (SAEM) algorithm combined with a Markov Chain Monte Carlo (MCMC) procedure. Several models (proportional, additive, mixed, logit) were investigated to describe the residual variability (ε). The between-subject variabilities (η or BSVs) were assumed to be exponential. The Bayesian information criterion (BIC) was used to select the best model. It is based on both the likelihood function and the number of model parameters and is more conservative than the Akaike Information Criterion.

A model including exponential functions of age corresponding to 3 phases (a, b and c) was used to fit the observations (Eq. 1):

1 $$ HT={HT}_{birth}+\left({HT}_{max}-{HT}_{birth}\right)\ast \left( HTa+ HTb+ HTc\right) $$

- from birth to age *A1*

$$ HTa= fa\ast \left(1-\mathit{\exp}\left(-0.693\ast t/A50a\right)\right)\kern1.25em \mathrm{with}\ \mathrm{t}=\mathrm{age} $$

b) from age *A1* to age *A2*

$$ HTb= fb\ast \left(1-\mathit{\exp}\left(-0.693\ast t/A50b\right)\right)\kern1.25em \mathrm{with}\ \mathrm{t}=\mathrm{age}-A1 $$

c) above age *A2*

$$ HTc= fc\ast \left(1-\mathit{\exp}\left(-0.693\ast t/A50c\right)\right)\kern1.25em \mathrm{with}\ \mathrm{t}=\mathrm{age}-A2 $$

where *HT*_*birth*_ is the birth length; *HT*_*max*_ the maximum (adult) height; *fa, fb* and *fc* the fractions of adult height gained at each phase; *A50a, A50b* and *A50c* the time durations in each phase for which 50% of *HTa, HTb* or *HTc* are reached; and, *A1* the age bounds between phases 1 and 2, and *A2* between 2 and 3. Normalizing *HT* by 180 cm, i.e., y = *HT*/180, the residual variability could be set to a logit model, and the resulting dependent variable, i.e., the relative height, was bound between 0 and 1.

The covariates of interest were: sex, ART regimen (dual nucleoside reverse transcriptase inhibitor (NRTI)-, NNRTI- or protease inhibitor (PI)-based), CD4 percentage, HIV-RNA load and CDC stage at ART initiation (baseline) and the occurrence of AIDS-defining events. A covariate was finally retained if i) its effect was biologically plausible, ii) a reduction in BIC value was observed and iii) it produced a reduction in the variability of the parameter, as assessed by the associated inter-subject variability. Graphical evaluation of the goodness-of-fit was performed using observed versus predicted height (PRED) and weighted residuals versus time and/or weighted residuals versus PRED. The final population model was evaluated by the normalized prediction distribution errors (NPDE) metrics [39] and the prediction-corrected visual predictive check (VPC) [40]. Diagnostic graphics and distribution statistics were obtained using RfN (link on http://wfn.sourceforge.net) via the R program.

*HT* versus age time-courses were simulated from their respective final population model and compared with the observed data to evaluate the predictive performance of the model. The vector of parameters from 100 replicates of the database was simulated using the final model. Each vector parameter was drawn from a distribution with a variance corresponding to the previously estimated BSV. A simulated residual error was added to each simulated variable. The 25th, 50th and 75th percentiles of the simulated dependent variables at each time were then overlaid on the observed data and a visual inspection was performed.

## Results

### Characteristics at ART initiation and last follow-up visit

The data from 477 children were included in this analysis. Table 1 presents their characteristics at ART initiation. Two-hundred and six (43%) males. Median age was 6.2 years (interquartile range (IQR), 1.8 to 9.6), with 65 (14%) under one year, 54% were in CDC stage B or C. Of the 477 children, 77 (16%) were underweight (WAZ < − 2), 235 (49%) had stunting (HAZ < − 2), and 32 (7%) experienced wasting (WHZ < − 2). Forty-one children (9%) had started therapy with dual NRTI regimens (before 2003) then switched to triple combination when it became widely available in Thailand, 79% children started on NNRTI-based regimen and 12% on PI. Of note, stunted children were significantly older at ART initiation, more often on NNRTI, in CDC stage B or C, with lower CD4 percentage and more likely to be underweight (Table 1).

**Table 1:** Baseline characteristics of 477 HIV-infected children

N (%) or median [IQR]	All	Height-for-age z-score at ART initiation				*p*-value
		<−3 SD	−3 to < − 2 SD	− 2 to < − 1 SD	≥ − 1 SD	
All	477	116 (24%)	119 (25%)	119 (25%)	123 (26%)	
Male	206 (43%)	56 (48%)	55 (46%)	43 (36%)	52 (42%)	0.25^a^
Age (years)	6.2 [1.8–9.6]	7.7 [5.1–10.6]	7.4 [3.9–9.9]	5.6 [1.5–9.5]	2.0 [0.8–6.5]	< 0.001^b^
ART regimen						< 0.001^a^
Dual NRTI-based regimen	41 (9%)	1 (1%)	5 (4%)	15 (13%)	20 (16%)	
PI-based regimen	57 (12%)	5 (4%)	8 (7%)	16 (13%)	28 (23%)	
NNRTI-based regimen	379 (79%)	110 (95%)	106 (89%)	88 (74%)	75 (61%)	
CD4 percentage	9 [2–17]	5 [1–11]	4 [1–13]	11 [4–18]	16 [10–23]	< 0.001^b^
HIV-RNA load (log_10_copies/mL)	5.17 [4.69–5.67]	5.16 [4.74–5.59]	5.20 [4.66–5.62]	5.13 [4.82–5.55]	5.22 [4.41–5.89]	0.98^b^
CDC HIV classification stage						< 0.001^a^
N	87 (18%)	12 (10%)	15 (13%)	20 (17%)	40 (33%)	
A	136 (28%)	25 (22%)	32 (27%)	41 (34%)	38 (31%)	
B	132 (28%)	34 (29%)	32 (27%)	38 (32%)	28 (23%)	
C	17 (26%)	45 (39%)	40 (33%)	20 (17%)	17 (23%)	
Weight-for-age z-score						< 0.001^a^
< −3 SD	18 (4%)	9 (8%)	7 (6%)	1 (1%)	1 (1%)	
− 3 to <−2 SD	59 (12%)	33 (28%)	12 (10%)	12 (10%)	2 (2%)	
− 2 to <−1 SD	196 (41%)	66 (57%)	68 (57%)	46 (39%)	16 (13%)	
≥ 1 SD	204 (43%)	8 (7%)	32 (27%)	60 (50%)	104 (84%)	
Weight-for-height z-score						0.72^a^
< −3 SD	5 (1%)	2 (2%)	2 (2%)	1 (1%)	–	
– 3 to <−2 SD	27 (6%)	7 (6%)	9 (7%)	6 (5%)	5 (4%)	
– 2 to <−1 SD	95 (20%)	27 (23%)	25 (21%)	22 (18%)	21 (17%)	
≥ −1 SD	350 (73%)	80 (69%)	83 (70%)	90 (76%)	97 (79%)	

*CDC* Centers for Disease Control and Prevention, *IQR* interquartile range, *N* number of children in category

^a^ Chi-square test

^b^ Kruskal-Wallis test

Over a median duration of 6.3 years (IQR, 3.0 to 8.3) of ART, 58 (12%) children developed at least one AIDS-defining events (ADEs), 29 (6%) children died, 92 (19%) were lost to follow-up, and 101 (21%) were referred to other hospitals. At the last follow-up visit, 52 (11%) of children presented underweight, 125 (26%) stunting, and 32 (7%) wasting. There was no association between the occurrence of an ADE and HAZ at last visit (*p* = 0.31) (Table 2). We found that HAZ at baseline was significantly different between children who had died, were lost to follow-up, or referred compared to those who completed the study (see Additional file 2: Table S3).

**Table 2:** Association between Height-for-age z scores at last visit and AIDS-defining events

AIDS-defining events	All	Height-for-age z-score at last follow-up visit				*p*-value
		<−3 SD	-3 to < −2 SD	-2 to < − 1 SD	≥ − 1 SD	
No	419 (88%)	41 (82%)	63 (84%)	111 (89%)	204 (90%)	0.31^a^
Yes	58 (12%)	9 (18%)	12 (16%)	14 (11%)	23 (10%)	

^a^ Chi-square test

Forty-eight HIV-infected children (16 males and 32 females) had height measurements after 18 years. Their median final height was 167 cm for boys, around the 28th percentile of the Thai norms (median = 170), and 154 cm for girls, around the 30th percentile of the Thai norms (median = 158). Finally, the proportion of stunted children decreased from 49% at ART initiation to 26% at last visit. The proportions of stunting were 25% (59/239) in children who started ART ≤6.2 years of age and 28% (66/238) in those who started after (*p*-value = 0.012).

### Model

**Fig. 1:**
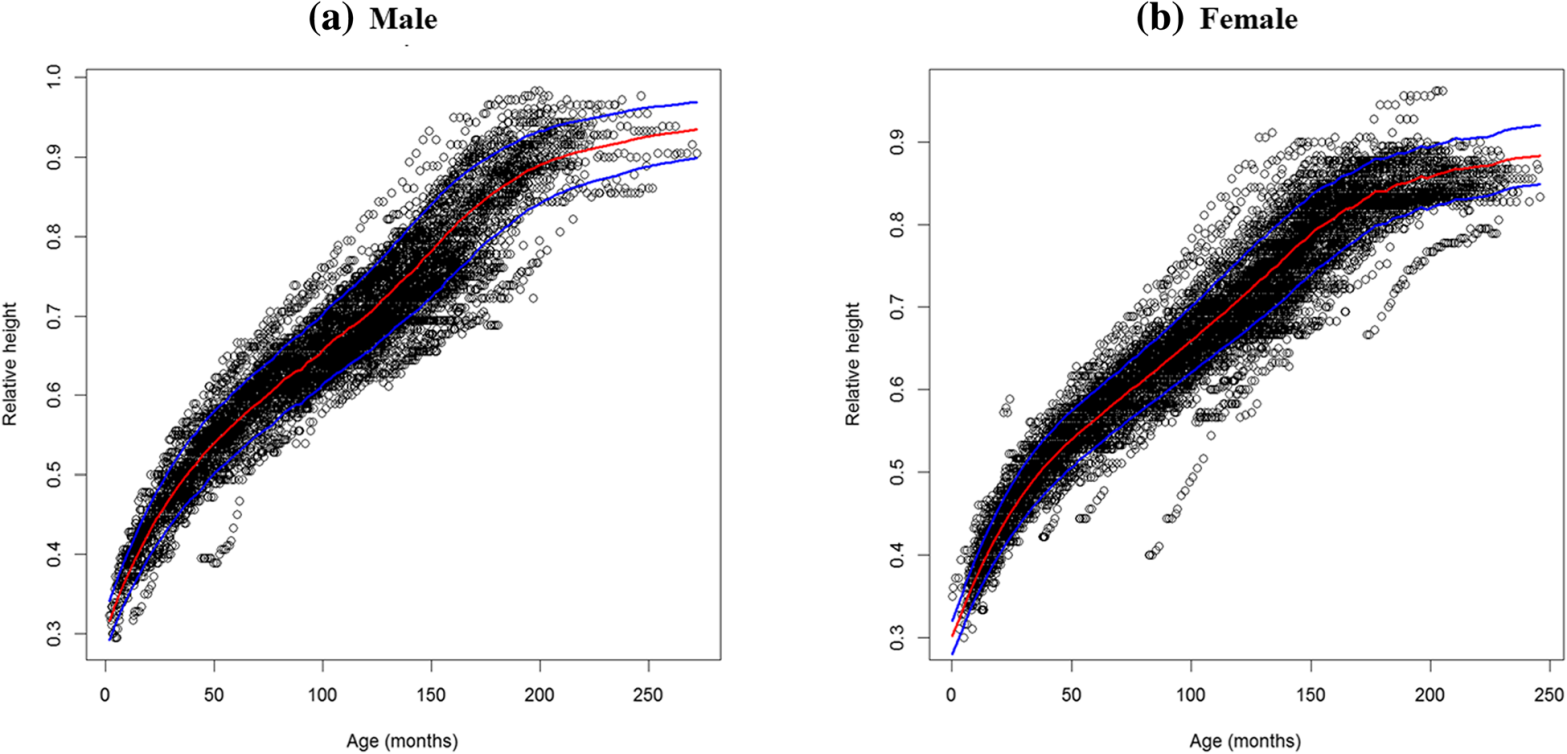
Height, normalized for 180 cm, as a function of age (**a**) male (**b**) female: the red curve stands for the median and the blue curves for the 25th, 75th percentiles, obtained from 100 simulations of the final model. Height measurements on the Y axis are divided by180 cm

A total of 18,596 height measurements were available, i.e. a median of 121 height measurements per child (IQR = 105–139). The basic model (Eq. 1) described satisfactorily the data. When a constant residual variability was used, the predicted-observed (PRED-OBS) plots both of males and females were acceptable but the VPC showed an over estimation of the variability. Normalizing height by 180 cm, i.e., y = Height/180, the residual variability could be set to a logit model, and the resulting dependent variable, i.e., the relative height, was bound between 0 and 1. This improved the fit and the VPC (Fig. 1). Type of ART regimen, CDC stage, CD4%, and HIV-RNA load at baseline were not associated with the maximum height (see Additional file 3: Table S4).

### Effect of other variables on the model

Adding the age at ART initiation, *Age*_*ART*_, improved the fit, showing an inverse association between height-growth velocity and age at ART initiation (if *Age*_*ART*_ is higher, the *A50a*, *A50b* or *A50c* parameters decrease by a *b1*, *b2* or *b3* fraction)*,* i.e. the older the child at ART initiation, the slower the growth.

The final model was

$$ {\displaystyle \begin{array}{cc}\mathrm{if}\ \left({Age}_{ART}<A 1\right)& b 1\;\mathrm{estimated}\ \left(\mathrm{if}\ \mathrm{not}\right)b 1=1\\ {}\mathrm{if}\ \left({Age}_{ART}>A 1\;\mathrm{and}\; Age(ART)<\mathrm{A}2\right)& b 2\;\mathrm{estimated}\ \left(\mathrm{if}\ \mathrm{not}\right)\ b 2=1\\ {}\mathrm{if}\ \left({Age}_{ART}>A 2\right)& b 3\;\mathrm{estimated}\ \left(\mathrm{if}\ \mathrm{not}\right)b 3=1\end{array}} $$

The Additional file 1: Table S1 and S2 provides the estimated values of the model parameters separately for males and females. The parameters were accurately estimated, as shown by the precision of these parameters (small relative standard errors).

**Fig. 2:**
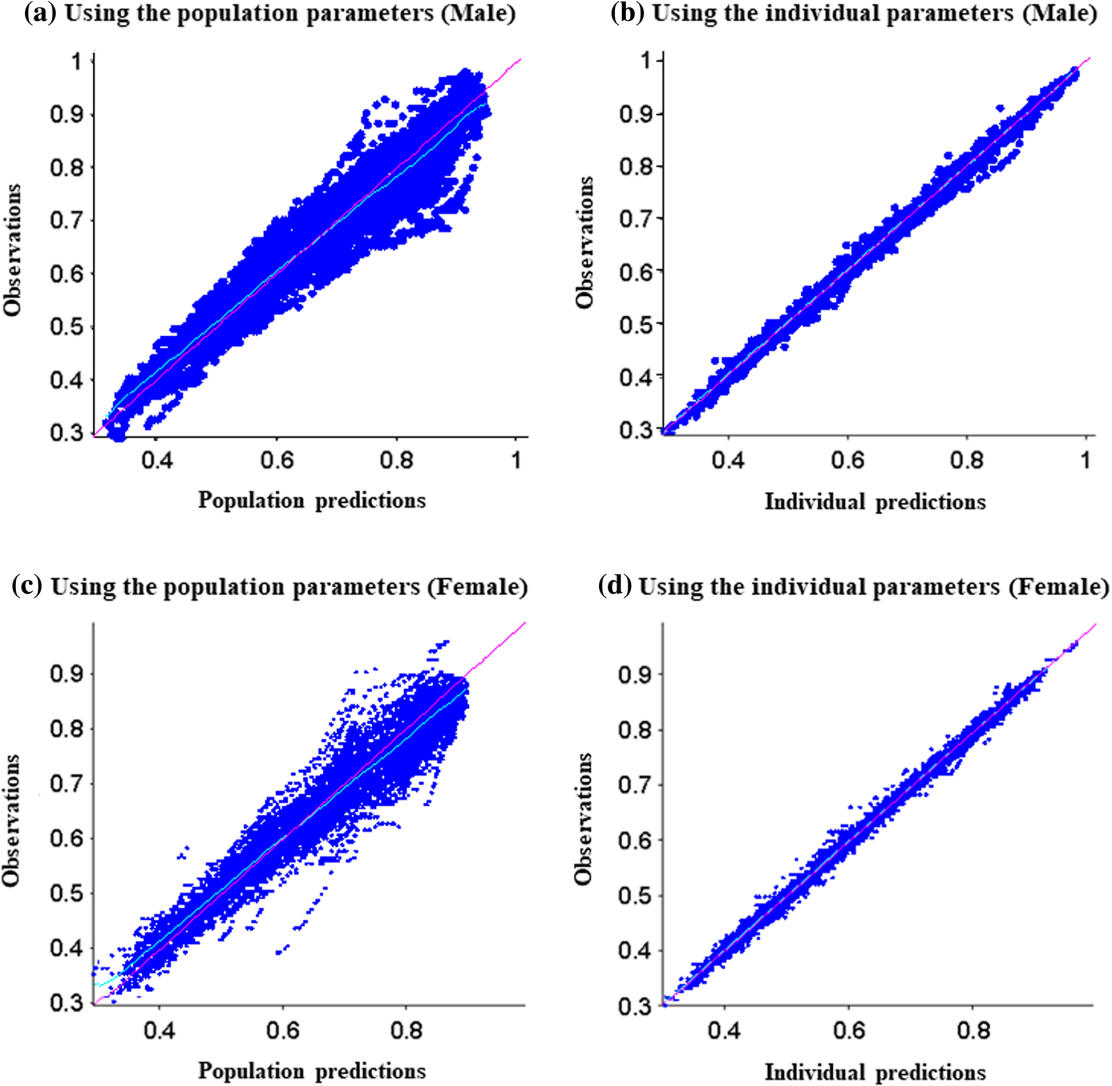
Relative observed versus predicted height from the population and individual models fits (**a**) population parameters among males (**b**) individual parameters among males (**c**) population parameters among females and (**d**) individual parameters among females: Y axis value 1 stands for 180 cm

The models showed a significant association between a final adult height (*HT*_*max*_) and the occurrence of ADEs both in males (178 versus 169 cm in those without and with ADEs, *p* < 0.01) and females (165 versus 159 cm, p < 0.01) (see Additional file 1: Table S1 and S2). Age at ART initiation, type of ART regimen, CDC stage, CD4%, and HIV-RNA load at baseline were not associated with the maximum height. Using either population or individual parameters from the final model, the heights predictions were very good (Fig. 2).

## Discussion

Based on repeated height measurements of a large number of HIV infected children in Thailand, we developed a new model to describe the patterns of height evolution. The best model included three phases from birth to final adult height. This model showed that height-growth velocity was greater in children who initiated therapy at earlier age, independently of CD4 level and CDC HIV Classification Stage. However, the final adult height (*HT*_*max*_) was associated with the occurrence of ADEs but not the age at ART initiation.

In this study we show that the time to reach 50% of each phase fractional height increased with the age at ART initiation. This is consistent with previous studies [8, 41–47], indicating that ART initiation at an early age significantly increased the height-growth velocity. Also, the maximal height (*HT*_*max*_) was related to the child’s sex –as in the general population.

Interestingly, height-growth velocity did not differ according to the initial ART regimen, even if it was a dual NRTI regimen as compared to a triple combination. Other studies did not find any difference of HAZ [8, 11] and weight-growth velocity [41, 48] according to the type of ART regimen. Also, the immunological or virological status at baseline had no impact on the height-growth velocity. However, the vast majority of children of this cohort started ART with significantly low CD4 percentages (median 9%) and it is unclear if this would apply to children starting ART with higher CD4 percentages. Unfortunately, some variables that can affect final height such as gestational age at birth, adherence, nutrition, exercise, puberty stage and genetic factors (e.g. parental heights) [49–51], were not available for the analysis.

A strength of our study is the large number of height measurements per children allowing us to develop a height-growth model that took into account the repeated measures in each individual. It has been shown that the height catch-up was improved when ART was initiated at an earlier age and in children with higher baseline HAZ z-score [52]. Most studies described improvements in HAZ [53, 54], height gain or even percentage increase [25, 55, 56] in relation to ART. In one study in Asian children among 273 perinatally HIV-infected adolescents [median age at ART initiation, 11.4 years], 19% were stunted according to the Thai child growth reference and half of those remained stunted over time [57]. However, in most studies, the follow-up duration was relatively short, and only one study in Spain examined the height evolution after a median follow up of 71 months [58].

In our study the type of initial ART regimen, NNRTI, PI or dual NRTI-based therapy, was not associated with the final height. In most of the studies in high-income countries, protease inhibitor (PI)-based regimens were used [26, 28, 41] in contrast to low and middle income countries where NNRTI were primarily used as first-line ART regimen [59, 60] following the World Health Organization (WHO) recommendations [61].

The fact that the occurrence of ADEs decreased the final height indicates that morbid episodes may slow-down children’s growth and emphasizes the need for close ART monitoring and adherence support.

There were some limitations in our study. Since females generally attain their final adult height at a younger age than males (around 15 years in females as compared to around 18 years in males). The small number of children with height measures after 18 years of age was a limitation. Indeed, 87 females had been measured after 15 years but only 16 males after 18 years, which impacted the precision of predictions beyond this age. Since changes in CD4 percentage and HIV-RNA load as well as in treatment may be related to the height velocity or final height, it could be more accurate to develop more complex models including time-dependent variables. Finally, there was one girl with endocrine disorder which could impact her growth. However, her growth curve was not affected by her disorder.

## Conclusions

Our results show the beneficial effect of age at ART initiation on the height-growth velocity but regardless of age at ART initiation and initial ART regimen, children were able to catch up in terms of final adult height. This supports the World Health Organization guidelines for widespread ART treatment of all HIV-infected children as soon as they are diagnosed [61].

## Additional files

Additional file 1: **Table S1.** Population parameters of height versus age model for 206 HIV-1-infected male children. **Table S2.** Population parameters of height versus age model for 271 HIV-1-infected female children. (DOCX 32 kb)

Additional file 2: **Table S3.** Comparison of baseline characteristics by follow-up status. (DOCX 19 kb)

Additional file 3: **Table S4.** Specific males’ models. **Table S5.** Specific females’ models. (DOCX 25 kb)

## Data Availability

This study used data from the ‘Prevention and Treatment of HIV infection and virus-associated cancers in Southeast Asia (PHPT)’ research unit, Chiang Mai, Thailand. Request for using these data should be addressed to the corresponding author.

## Abbreviations

ADE: AIDS-defining event
AIC: Akaike information criterion
ART: Antiretroviral treatment
BIC: Bayesian information criterion
BSV: Between-subject variability
CDC: Centers for Disease Control and Prevention
HAZ: Height-for-age z-score
IQR: Interquartile range
LRT: Likelihood ratio test
MCMC: Markov Chain Monte Carlo
NNRTI: Non-nucleoside reverse transcriptase inhibitor
NPDE: Normalized prediction distribution errors
NRTI: Nucleoside reverse transcriptase inhibitor
PHPT: Program for HIV Prevention and Treatment
PI: Protease inhibitor
PRED: Predicted height
SAEM: Stochastic approximation expectation maximization
VPC: Visual predictive check
WAZ: Weight-for-age z-score
WHO: World Health Organization
WHZ: Weight-for-height z-score
