# Supplementary material for: Impact of antiretroviral treatment on height evolution of HIV infected children
Source: BMC Pediatr. 2019 Aug 17;19:287. doi: 10.1186/s12887-019-1663-8 (PMC6697969; doi:10.1186/s12887-019-1663-8)
Supplement: Supplementary file 1 — Table S1. Population parameters of height versus age model for 206 HIV-1-infected male children. Table S2. Population parameters of height versus age model for 271 HIV-1-infected female children. (DOCX 32 kb) [file 12887_2019_1663_MOESM1_ESM.docx]

**Table S1**. Population parameters of height versus age model for 206 HIV-1-infected male children.

| **Parameters** | **Mean** | **Relative standard error (%)** |
| --- | --- | --- |
| *Structural model* |  |  |
| *HT_birth_* | 54.7 | 1 |
| *HT_max_* | 178 | 0 |
| *fa* | 0.557 | 1 |
| *fb* | 0.274 | 2 |
| *A50a* | 50.8 | 2 |
| *A50b* | 75.3 | 4 |
| *A50c* | 15.7 | 11 |
| *A1* | 73.5 | 2 |
| *A21* | 69.9 | 2 |
| *b1* | 0.713 | 2 |
| *b2* | 0.727 | 4 |
| *b3* | 0.676 | 9 |
| *HT_max_*, cm (if ADEs) | -9.31 | 32^†^ |
| *Statistical model* |  |  |
| ω *HT_birth_* | 6.48 | 6 |
| ω *HT_max_* | 8.54 | 6 |
| ω *fa* | 0.240 | 6 |
| ω *fb* | 0.350 | 7 |
| ω *A50a* | 0.287 | 6 |
| ω *A50b* | 0.410 | 7 |
| ω *A50c* | 1.010 | 7 |
| ω *A1* | 0.273 | 6 |
| ω *A2*1 | 0.235 | 8 |
| ω *b1* | 0.442 | 11 |
| ω *b2* | 1.200 | 9 |
| ω *b3* | 1.210 | 15 |
| Structural model by group |  |  |
| *HT_max_*: Male without ADEs | 178 | 0 |
| *HT_max_*: Male with ADEs | 169 | 2 |

AIDs, AIDS-defining events

^†^ p-value <0.01

*fc* = 1 - *fa* - *fb* = 1 - 0.557 - 0.274 = 0.169;

*A2* = *A1* + *A21* = 73.5 + 69.9 = 143.4;

RSE%, relative standard error (standard error of estimate / estimate*100);

*ω*, Inter-individual variability.

**Table S2**. Population parameters of height versus age model for 271 HIV-1-infected female children.

| **Parameters** | **Mean** | **Relative standard error (%)** |
| --- | --- | --- |
| *Structural model* |  |  |
| *HT_birth_* | 53.6 | 1 |
| *HT_max_* | 165 | 0 |
| *fa* | 0.576 | 1 |
| *fb* | 0.270 | 2 |
| *A50a* | 45.8 | 2 |
| *A50b* | 57.4 | 3 |
| *A50c* | 13.8 | 6 |
| *A1* | 69.2 | 2 |
| *A21* | 58.7 | 2 |
| *b1* | 0.678 | 2 |
| *b2* | 0.560 | 4 |
| *b3* | 0.999 | 0 |
| *HT_max_*, cm (if ADEs) | -6.10 | 38^†^ |
| *Statistical model* |  |  |
| ω *HT_birth_* | 5.58 | 5 |
| ω *HT_max_* | 8.87 | 5 |
| ω *fa* | 0.159 | 6 |
| ω *fb* | 0.282 | 6 |
| ω *A50a* | 0.250 | 5 |
| ω *A50b* | 0.449 | 6 |
| ω *A50c* | 0.716 | 6 |
| ω *A1* | 0.245 | 5 |
| ω *A2*1 | 0.245 | 5 |
| ω *b1* | 0.487 | 9 |
| ω *b2* | 0.802 | 9 |
| ω *b3* | 0.633 | 24 |
| Structural model by group |  |  |
| *HT_max_*: Female without ADEs | 165 | 0 |
| *HT_max_*: Female with ADEs | 159 | 1 |

ADEs, AIDS-defining events

^†^ p-value <0.01

*fc* = 1 - *fa* - *fb* = 1 – 0.576 – 0.270 = 0.154;

*A2* = *A1* + *A21* = 69.2 + 58.7 = 127.9;

RSE%, relative standard error (standard error of estimate / estimate*100);

*ω*, Inter-individual variability.

A model including exponential functions of age corresponding to 3 phases (a, b and c) fitted the observations (Equation 1):

*HT = HT_birth_ + (HT_max_ – HT_birth_)*(HTa + HTb + HTc)* (1)

a) from birth to age *A1*

*HTa = fa*(1 – exp(-0.693*t/(A50a*b1)))* with t = age

b) from age *A1* to age *A2*

*HTb = fb*(1 – exp(-0.693*t/(A50b*b2)))* with t = age – *A1*

c) above age *A2*

*HTc = fc*(1 – exp(-0.693*t/(A50c*b3)))* with t = age – *A2*

where *HT_birth_* is the birth length; *HT_max_* the maximum (adult) height; *fa, fb* and *fc* the fractions of adult height gained at each phase; *A50a, A50b* and *A50c* the time durations in each phase for which 50% of *HTa, HTb* or *HTc* are reached; and, *A1* the age bounds between phases 1 and 2, and *A2* between 2 and 3.

Adding the age at ART initiation, *Age_ART_*, improved the fit, showing an inverse association between height-growth velocity and age at ART initiation (if *Age_ART_* is higher, the *A50a*, *A50b* or *A50c* parameters decrease by a *b1*, *b2* or *b3* fraction)*, i.e*. the older the child at ART initiation, the slower the growth.

The final model was

if (*Age_ART_* <*A1*) *b1* estimated (if not) *b1*= 1

if (*Age_ART_* >*A1* and *Age(ART)* <A2) *b2* estimated (if not*) b2* = 1

if (*Age_ART_* >*A2*)  *b3* estimated (if not) *b3* = 1

**Example of prediction**

|  | **Sex** | **ADEs** | **Age at ART initiation**  **(months)** | **Age at last height measurement**  **(months)** | **Observed height**  **(cm)** | **Predicted height**  **(cm)** |
| --- | --- | --- | --- | --- | --- | --- |
| 1 | Male | No | 63.179 | 172.682 | 162 | 156.194 |
| 2 | Male | Yes | 9.692 | 149.257 | 137 | 134.827 |
| 3 | Female | No | 120.739 | 172.485 | 158.9 | 155.208 |
| 4 | Female | Yes | 139.203 | 211.023 | 150 | 151.124 |

For example, the calculation for case 1 was as follows:

HTa = 0.557 * (1 – exp(-0.693*172.268 /(50.8*0.713))) with t = 172.268 months

HTb = 0.274 * (1 – exp(-0.693*(98.768)/(75.3*1))) with t = 172.268 – 73.5 = 98.768 months

HTc = 0.169 * (1 – exp(-0.693*(28.868)/(15.7*1))) with t = 172.268 – 143.4 = 28.868 months

HT = 54.7 + (178 – 54.7) * (0.537 + 0.164 + 0.123) = 156.194
