# Supplementary material for: Impact of antiretroviral treatment on height evolution of HIV infected children
Source: BMC Pediatr. 2019 Aug 17;19:287. doi: 10.1186/s12887-019-1663-8 (PMC6697969; doi:10.1186/s12887-019-1663-8)
Supplement: Supplementary file 2 — Table S3. Comparison of baseline characteristics by follow-up status. (DOCX 19 kb) [file 12887_2019_1663_MOESM2_ESM.docx]

**Table S3.** Comparison of baseline characteristics by follow-up status

| N (%) or median [IQR] | **Follow-up status** | | **p-value** |
| --- | --- | --- | --- |
|  | **Death/LTFU/** **Refer**  **(n=222)** | **Complete**  **(n=255)** |  |
| Male | 101 (46%) | 105 (41%) | 0.342^a^ |
| Age (years) | 6.9 [2.0-10.1] | 5.8 [1.6-9.3] | 0.064^b^ |
| ART regimen |  |  | 0.391^a^ |
| Dual NRTI-based regimen | 21 (9.5%) | 20 (8%) |  |
| PI-based regimen | 22 (9.9%) | 35 (14%) |  |
| NNRTI-based regimen | 179 (80.6%) | 200 (78%) |  |
| CD4 percentage | 8 [2-18] | 9 [2-17] | 0.534^b^ |
| HIV-RNA load (log_10_copies/mL) | 5.12 [4.64-5.60] | 5.23 [4.75-5.73] | 0.254^b^ |
| CDC HIV classification stage |  |  | 0.489^a^ |
| N | 40 (18%) | 47 (18%) |  |
| A | 63 (28%) | 73 (29%) |  |
| B | 30 (31%) | 64 (25%) |  |
| C | 51 (23%) | 71 (28%) |  |
| Height-for-age z-score |  |  | 0.002^a^ |
| <-3 SD | 34 (15%) | 16 (6%) |  |
| -3 to <-2 SD | 41 (18%) | 34 (13%) |  |
| -2 to <-1 SD | 50 (23%) | 75 (30%) |  |
| ≥1 SD | 97 (44%) | 130 (51%) |  |
| Weight-for-age z-score |  |  | 0.079^a^ |
| <-3 SD | 7 (3%) | 6 (2%) |  |
| -3 to <-2 SD | 20 (9%) | 19 (7%) |  |
| -2 to <-1 SD | 80 (36%) | 68 (27%) |  |
| ≥1 SD | 115 (52%) | 162 (64%) |  |
| Weight-for-height z-score |  |  | 0.079^a^ |
| <-3 SD | 5 (2%) | 1 (1%) |  |
| -3 to <-2 SD | 11 (5%) | 5 (2%) |  |
| -2 to <-1 SD | 27 (12%) | 31 (12%) |  |
| ≥-1 SD | 179 (81%) | 218 (85%) |  |

CDC, Centers for Disease Control and Prevention; IQR, interquartile range; n, number of children in category; LTFU, Loss to follow-up

^a^ Chi-square test

^b^ Kruskal-Wallis test
