# Supplementary material for: Impact of antiretroviral treatment on height evolution of HIV infected children
Source: BMC Pediatr. 2019 Aug 17;19:287. doi: 10.1186/s12887-019-1663-8 (PMC6697969; doi:10.1186/s12887-019-1663-8)
Supplement: Supplementary file 3 — Table S4. Specific males’ models. Table S5. Specific females’ models. (DOCX 25 kb) [file 12887_2019_1663_MOESM3_ESM.docx]

**Table S4.** Specific males’ models.

| **Males’ models** | **-2LL** | **AIC** | **BIC** | | **BIC_COVARIATE_ - BIC_REFERENCE_^a^** | **p-value^b^** |
| --- | --- | --- | --- | --- | --- | --- |
| 1) REFERENCE, covariate-free | -45393 | -45343 | -45261 | | NA | NA |
| Covariate on *HT_birth_*^c^ |  |  |  | |  |  |
| 2) + CDC HIV classification stage | -38786 | -38734 | -38649 | | +6612 | NA |
| 3) +CD4 percentage at baseline | -45037 | -44985 | -44900 | | +361 | NA |
| 4) +HIV-RNA load at baseline | -42419 | -42367 | -42281 | | +2980 | NA |
| 5) +Type of ART regimen | -45901 | -45849 | -45763 | -502 | | 0.110 |
| Covariate on *HT_max_*^d^ |  |  |  | |  |  |
| 6) + CDC HIV classification stage | -46356 | -46304 | -46218 | | -957 | 0.370 |
| 7) +CD4 percentage at baseline^e^ | -46468 | -46416 | -46331 | | -1070 | 0.015 |
| 8) +HIV RNA at baseline | -43888 | -43836 | -43750 | | +1511 | NA |
| 9) +Type of ART regimen | -38497 | -38445 | -38360 | +6901 | | NA |
| 10) +ADEs | -45701 | -45649 | -45563 | | -302 | 0.002 |
| Covariates on *HT_birth_* and *HT_max_* |  |  |  | |  |  |
| 11) +CD4 & ADEs on *HT_max_* | -67531 | -67477 | -67380 | | -22119 | 0.340 |

ADEs, AIDS-defining events; CDC, Centers for Disease Control and Prevention; NA, not applicable

A covariate was finally retained if i) its effect was biologically plausible, ii) a reduction in BIC value was observed and iii) it produced a reduction in the variability of the parameter, as assessed by the associated inter-subject variability.

^a^ A negative BIC difference indicates that the addition of the specified covariate significantly improves the model

^b^ p-value after adding covariates

^c^ Height at birth

^d^ Maximum adult height

^e^ CD4 percentages at baseline was considered as non-significant. It was associated with height at adult age only for males which is not biologically plausible.

**Table S5.** Specific females’ models.

| **Females’ models** | **-2LL** | **AIC** | **BIC** | **BIC_COVARIATE_ - BIC_REFERENCE_^a^** | **p-value^b^** |
| --- | --- | --- | --- | --- | --- |
| 1) REFERENCE, covariate-free | -67538 | -67488 | -67398 | NA | NA |
| Covariate on *HT_birth_*^c^ |  |  |  |  |  |
| 2) + CDC HIV classification stage | -67267 | -67215 | -67122 | + 275 | NA |
| 3) +CD4 percentage at baseline | -66676 | -66624 | -66531 | + 867 | NA |
| 4) +HIV-RNA load at baseline | -67098 | -67046 | -66952 | + 445 | NA |
| 5) +Type of ART regimen | -66049 | -65997 | -65903 | +1495 | NA |
| Covariate on *HT_max_*^d^ |  |  |  |  |  |
| 6) + CDC HIV classification stage | -67168 | -67116 | -67023 | + 375 | NA |
| 7) +CD4 percentage at baseline | -67085 | -67033 | -66939 | + 458 | NA |
| 8) +HIV RNA at baseline | -67161 | -67109 | -67016 | + 458 | NA |
| 9) +Type of ART regimen | -64905 | -64853 | -64759 | +2639 | NA |
| 10) +ADEs | -68361 | -68309 | -68216 | - 816 | 0.009 |

ADEs, AIDS-defining events; CDC, Centers for Disease Control and Prevention; NA, not applicable

A covariate was finally retained if i) its effect was biologically plausible, ii) a reduction in BIC value was observed and iii) it produced a reduction in the variability of the parameter, as assessed by the associated inter-subject variability.

^a^ A negative BIC difference indicates that the addition of the specified covariate significantly improves the model

^b^ p-value after adding covariates

^c^ Height at birth

^d^ Maximum adult height
